# Workplace and non-workplace mild traumatic brain injuries in an outpatient clinic sample: A case-control study

**DOI:** 10.1371/journal.pone.0198128

**Published:** 2018-06-01

**Authors:** Douglas P. Terry, Grant L. Iverson, William Panenka, Angela Colantonio, Noah D. Silverberg

**Affiliations:** 1 Department of Physical Medicine and Rehabilitation, Harvard Medical School, Boston, Massachusetts, United States of America; 2 Spaulding Rehabilitation Hospital, Boston, Massachusetts, United States of America; 3 MassGeneral Hospital for Children^™^ Sports Concussion Program, Boston, Massachusetts, United States of America; 4 Home Base, A Red Sox Foundation and Massachusetts General Hospital Program, Boston, Massachusetts, United States of America; 5 British Columbia Neuropsychiatry Program, Vancouver, British Columbia, Canada; 6 Department of Psychiatry, University of British Columbia, Vancouver, British Columbia, Canada; 7 Department of Occupational Science and Occupational Therapy, University of Toronto, Toronto, Ontario, Canada; 8 Toronto Rehabilitation Institute, University Health Network, Toronto, Ontario, Canada; 9 Division of Physical Medicine & Rehabilitation, University of British Columbia, Vancouver, British Columbia, Canada; 10 Rehabilitation Research Program, Vancouver Coastal Health Research Institute, Vancouver, British Columbia, Canada; Instituto Cajal-CSIC, SPAIN

## Abstract

Individuals who are injured in the workplace typically have a greater risk of delayed return to work (RTW) and other poor health outcomes compared to those not injured at work. It is not known whether these differences hold true for mild traumatic brain injuries (MTBI). The present study examined differences associated with workplace and non-workplace MTBI upon intake to a specialty MTBI clinic, their outcomes, and risk factors that influence RTW. Slow-to-recover participants were recruited from consecutive referrals to four outpatient MTBI clinics from March 2015 to February 2017. Two clinics treat Worker’s Compensation claimants and two clinics serve patients with non-work related injuries in the publically funded health care system. Of 273 eligible patients, 102 completed an initial study assessment (M age = 41.2 years, SD age = 11.7; 54% women) at an average of 2–3 months post injury. Participants were interviewed about their MTBI and completed a battery of standardized questionnaires and performance validity testing. Outcomes, including RTW, were assessed via telephone follow-up 4–5 months later. Workplace injuries comprised 45.1% of the sample. The workplace MTBI group had a greater proportion of men and lower education levels compared to the non-workplace MTBI group. The two groups had a comparable post-concussion symptom burden and performance validity test failure rate. Workplace MTBI was associated with greater post-traumatic stress symptoms. Fifteen patients (14.7%) were lost to follow-up. There were no workplace/non-workplace MTBI differences in RTW outcome at 6–7 months post injury. Of the entire sample, 42.5% of patients had full RTW, 18.4% had partial RTW, and 39.1% had no RTW. Greater post-concussion symptom burden was most predictive of no RTW at follow-up. There was no evidence that the workplace and non-workplace MTBI groups had different risk factors associated with prolonged work absence. Despite systemic differences in compensation and health care access, the workplace and non-workplace MTBI groups were similar at clinic intake and indistinguishable at follow-up, 6–7 months post injury.

## Introduction

Approximately 1 in 4 mild traumatic brain injuries (MTBIs) in adults occur at work[1,2]. Across the spectrum of severity, workplace brain injuries are associated with substantial productivity loss, economic burden[3–5], persistent symptoms and occupational disability[1,6]. Considering traumatic injuries of all kinds, patients who are injured at their workplace are at greater risk of delayed return to work (RTW) and other poor health outcomes than those who were injured outside of work[7]. It is not known whether this difference holds for MTBI and which factors account for worse outcomes from workplace injuries. There are several reasons to expect differences between workplace and non-workplace MTBIs. For example, whereas many falls and recreational accidents are non-compensable, patients who are injured at work are legally entitled to compensation[8]. Compensation access is highly predictive of delayed RTW[9,10]. Worker’s Compensation claimants in Canada have substantial rates of suspected symptom exaggeration after MTBI[11,12]. However, compensation access cannot fully account for differences in RTW rates. For example, in New Zealand’s universal entitlement system, where workplace and non-workplace injures afford the same access to compensation, workplace injuries are associated with a greater risk of delayed RTW, as well as long-term physical and emotional health problems[7].

Differences in patient demographics might contribute to differences between workplace and non-workplace injuries. MTBI is more common in men[13], and this gender gap is wider for workplace injuries (including MTBI)[2,4,14,15], but women are at greater risk for chronic symptoms following MTBI[16–18]. Women more often access health services than men in some settings, but not for a workplace MTBI[19]. Gender and workplace factors may also interact. Traditionally masculine work environments may be less supportive for returning injured workers[20]. MTBI occurs disproportionately in manufacturing, construction, and transportation[2,4,6,15]. These occupations are associated with lower education attainment and decision-making latitude at work, factors that have been previously linked to worse outcomes from MTBI[21–24].

Another factor that may contribute to poor prognosis and delayed RTW after work injury is blame attribution[8,25,26]: who or what the patient perceives is at fault for their injury. Those who are injured at work are most likely to blame equipment or others in the workplace rather than themselves, whereas self-blame is most common in non-workplace injuries[8]. Blame perception regarding work injury is predictive of disability at 6-months post injury[25]. The broader concept of injustice may be more predictive of occupational disability than simply asking injured workers who is at fault[27–30]. In relation to an injury or illness, perceived injustice is a set of beliefs that comprise blame, a sense of unfairness, severity of loss, and that their loss is irreparable[30]. Injured workers who blame their employer may be less inclined to return to that work setting because they feel unsafe. Further, those with a workplace MTBI may face relationship strain related to the increased work burden on co-workers in their absence or feeling retaliated against by their employer/company[31].

Mental health and interpersonal/psychosocial difficulties may also be barriers to returning to work. Posttraumatic stress disorder (PTSD) is a relatively frequent (12–27%) comorbidity that complicates recovery from MTBI[32]. Even symptoms of post-traumatic stress that are not severe enough to meet full diagnostic criteria magnify the risk of not returning to work at 6–9 months post MTBI[21]. PTSD may be especially common and problematic following workplace MTBI, because workplace injuries in general are associated with more PTSD symptoms than non-workplace injuries and PTSD symptoms, in turn, are associated with delayed RTW[8]. They may be a particular barrier for returning to the same job with the same employer[33], because that would by definition involve exposing oneself to trauma-related triggers.

The primary aim of this study was to compare symptomatic workplace vs. non-workplace patients with MTBIs with respect to their initial presentation to a specialty MTBI clinic and RTW outcomes. A secondary aim was to determine if different risk factors influence RTW in each group. Compared to the non-workplace MTBI group, we predicted that the workplace MTBI group would include a greater proportion of men, have lower education levels, a lower proportion of occupations with high decision-making latitude, stronger perceived injustice beliefs, worse PTSD symptoms, higher rate of symptom invalidity, and lower rate of RTW. We further hypothesized that perceived injustice and traumatic stress symptoms would be more strongly related to RTW status at follow-up in the workplace MTBI group.

## Materials and methods

### Participants and procedures

Participants were recruited via consecutive referrals to four outpatient clinics in the Vancouver (Canada) area that specialize in MTBI/concussion rehabilitation from March 2015 to February 2017. Two of these clinics treat Worker’s Compensation claimants, while the other two serve patients with non-work related injuries, in the publicly funded health care system. Participants with work-related MTBIs were referred to clinic by their Worker’s Compensation Board case managers. Participants whose MTBI was not work-related could have been referred by their family physician or specialist, or self-referred. Referrals to these speciality MTBI clinics were initiated because of persistent symptoms or risk factors for persistent symptoms. Patients referred to these clinics are usually on an atypical recovery trajectory and they are considered at risk for long-term symptoms and problems. To be eligible for the study, participants had to be 18–65 years old, have sustained a MTBI within the past six months (based on the World Health Organization Neurotrauma Task Force definition[34]), be fluent in English, and employed prior to the injury. The University of British Columbia Ethics Board, the Vancouver Coastal Health Research Institute, and the Fraser Health Research Institute approved this study. Participation in this research study was voluntary. The measures described below were administered to patients as part of a research battery (i.e., not in usual clinical care). Of the 273 patients who agreed to be contacted for research purposes during the recruitment period, 93 were excluded based on an initial screening phone call (i.e., 44 did not meet MBTI criteria; 26 were injured > 6 months prior; 16 were not employed prior to the injury; 4 were not fluent in English; 3 were > 65 years old) and 40 declined to participate. 140 participants were invited for an initial assessment, but 38 could not be scheduled or did not attend. There were 102 participants who gave written consent for this study and completed the initial study assessment.

Participants were assessed at two time points, when they initially presented to clinic and again 4–5 months following their initial study visit, after undergoing rehabilitation. The initial study assessment occurred 2–26 weeks post injury (M = 12.05; SD = 6.26) and included a standardized semi-structured interview of their demographics (e.g., education, occupation), prior health history (e.g., pre-injury mental health treatment), and MTBI [e.g., self-reported injury characteristics, such as mechanism of injury, loss of consciousness (LOC), amnesia, confusion/disorientation, co-occurring orthopaedic injury, healthcare utilization, compensation-seeking status]. Based on the participant’s report of their injury, LOC was classified into one of the following mutually exclusive categories: witnessed (i.e., participant learned from another person’s eye witness account that they had an LOC), unwitnessed (i.e., participant inferred that they had an LOC but were not observed by another person to be unresponsive), unknown LOC (i.e., participant stated that they were unsure whether they had an LOC or not), or denied LOC. Medical records were not accessed for the purpose of this study. Further, participants completed several questionnaires (see Measures). The follow-up assessment occurred 21–47 weeks post injury (M = 31.57; SD = 6.05) via telephone. During this follow-up assessment, participants participated in a structured interview about their RTW status. Their answers were classified into three possible outcomes to form our primary outcome variable: (i) Full RTW: Returned to same hours and responsibilities at their pre-injury job, or started a new job with comparable hours/responsibilities; (ii) Partial RTW: Returned to pre-injury job but with reduced hours or responsibilities, or started a new job that is less demanding; or (iii) No RTW: Not working because of the MTBI, other injuries from the same event, or unrelated to injury event. Additionally, at the time of the follow-up assessment, a treating clinician at each site completed a health service utilization summary for each patient (6 summaries missing; 3 workplace MTBI and 3 non-workplace MTBI).

### Measures

#### Injustice Experience Questionnaire (IEQ)

The IEQ[27] is a 12-item questionnaire that assesses perceived injustice associated with injury. This is operationalized as a set of cognitions regarding how individuals view their injury and its consequences, including beliefs about being treated unfairly and suffering unnecessarily as a result of another’s actions. Individual items are rated on a 0–4 scale based on frequency (i.e., “never” to “always”) and summed for the total score (range: 0–48). This scale correlates with symptom chronicity and occupational disability in a variety of health conditions[27,28,30,35,36]. Internal consistency was excellent for this scale in the present sample (Cronbach’s α = .92).

#### PTSD checklist for DSM-5 (PCL-5)

The PCL-5 is a 20-item questionnaire that assesses symptoms of post-traumatic stress over the past month. Participants rate how much they were bothered by each symptom (0/“not at all” to 4/“extremely”). Total scores range from 0–80. This measure has high reliability and good evidence of convergent and discriminant validity[37,38]. Internal consistency was high for this scale in the present sample (Cronbach’s α = .94).

#### Brief Pain Questionnaire (BPQ)

The BPQ is a 5-item questionnaire that assesses current pain in the following five bodily regions: the head/skull, neck, chest/abdomen/back, arms/shoulders, and pelvis/legs. Current pain intensity is rated from 0 (none) to 3 (severe) in each bodily region, with a possible score range of 0–15. Internal consistency was acceptable at the initial (Cronbach’s α = .75) and follow-up assessments (Cronbach’s α = .79).

#### Medical Symptom Validity Test (MSVT)

Compensation-seeking status is strongly associated with outcome from MTBI, including RTW[9,39]. People who are involved in compensation claims are more likely to report persistent/exaggerated symptoms[40] and have poor effort during neuropsychological testing[41]. Performance validity testing[42] is a term used for purpose-built tests or embedded validity indicators in a neuropsychological evaluation. Performance validity test failure is independently associated with both compensation-seeking (vs. ineligible for compensation) and workplace (vs. non-workplace) MTBI[43]. The MSVT is sensitive to motivational factors but insensitive to the effects of genuine memory impairment and TBI.[44,45] We used evidence-based cut-off scores on the three “easy” subtests (Immediate Recognition, Delayed Recognition, and Consistency) to identify cases with probable below-capacity performance. The MSVT has been validated for use in patients with TBI and compares favorably with legacy instruments[46–49].

#### Self-prognosis rating

At the initial study assessment, participants were asked a single question about how they expected their recovery would progress. Similar self-prognosis ratings have been shown to have prognostic value in both MTBI[50–52] and mixed traumatic injury samples[53]. Participants selected whether they thought their symptoms would: “get better soon,” “get better slowly,” “never get better,” or “don’t know.” “Never get better” was a rarely endorsed response (n = 1), so this data point was excluded from analyses.

#### British Columbia Postconcussion Symptom Inventory (BC-PSI)

The BC-PSI is a 16-item self-report questionnaire that measures the frequency and severity of postconcussion symptoms. Item scores are derived from the ratings on frequency and severity scores. The total BC-PSI score is the sum of the item scores (range: 0–52). This scale’s reliability and validity have been well-established[54–56]. In our sample, internal consistency was high at the initial study assessment (Cronbach’s α = .96) and at follow-up (Cronbach’s α = .96). Similar to our previous studies, patients were classified as meeting *International Classification of Diseases*, *10*^*th*^ *Revision* (ICD-10) criteria for mild postconcussional syndrome (Mild PCS) if they reported symptoms as being mild or worse (i.e., item scores ≥2) in at least three symptom categories (i.e., physical symptoms, cognitive symptoms, emotional symptoms, or sleep-related symptoms)[57,58]. Moderate PCS (i.e., item scores ≥3 greater across three symptom categories) was calculated similarly.

#### World Health Organization Disability Assessment Schedule 2.0 (WHODAS)-12 item

The WHODAS is a brief, cross-cultural and disease non-specific standardized disability instrument[59]. It measures disability in six domains and is sensitive to a variety of medical and psychiatric conditions, including TBI[60–64]. We recently reported that this measure has desirable psychometric characteristics in MTBI, with a sample that overlaps with the present study[65]. Patients select how much difficulty they have experienced over the past 30 days (1/“none” to 5/“extreme or cannot do”, score range: 12–60). The structured interview format was administered in this study. In our sample, internal consistency was high (Cronbach’s α = .91).

#### Lam Employment Absence and Productivity Scale

The LEAPS is a questionnaire that assesses work difficulties and absenteeism[66]. On item 4, parts a-g, patients report how much they were bothered by problems like low energy, poor concentration, anxiety, and making mistakes while at work over the past two weeks (0/“none of the time, 0%” to 4/“all of the time, 100%”; score range: 0–28). A total score of 6 or higher indicates some degree of work impairment. In our sample, internal consistency was acceptable for this measure (Cronbach’s α = .78). As in a prior study[57], we used the LEAPS to add granularity to RTW outcome classifications.

### Statistical analyses

Independent samples t-tests were used to evaluate group differences on continuous variables between the workplace and non-workplace MTBI groups. Analyses of covariance (i.e., ANCOVAs) were used to compare the groups on measures with continuous variables (i.e., age, PCL-5, IEQ, BC-PSI, WHODAS, number of primary care visits) while statistically controlling for other continuous variables (i.e., weeks from injury to assessment) that may influence this relationship. A small number of patients omitted a single item on the BC-PSI (n = 1) and PCL-5 (n = 5). We imputed these missing values by averaging the participant’s responses to the remainder of the items from that scale and rounding the average item value to the nearest whole number. In so doing, a total of 0.07% (6 of 8,304) questionnaire items were imputed. We used chi-squared tests to compare the groups on categorical variables. All comparisons used a statistical significance level of *p*<.05.

Logistic regression was used to examine whether the prognostic value of previously established and novel candidate risk factors for delayed RTW after MTBI differed for participants with workplace vs. non-workplace MTBI. “Any RTW” (i.e., partial and full RTW) vs No RTW was the dichotomous outcome. Post-concussion symptom reporting[23,51], co-occurring orthopedic injuries[23,62], and self-rated prognosis[51,53] have been significant predictors in prior studies and so were included as predictors in our logistic regression analyses. Self-rated prognosis was treated categorically, with “get better soon” serving as the reference group. Traumatic stress (PCL-5) was included as a predictor because of preliminary evidence of its prognostic utility after MTBI for RTW[51] and prolonged symptoms[23]. Further, probable below-capacity performance (i.e., MSVT failure) was included as a predictor because lower PVT scores have been predictive of longer wage replacement benefits (a proxy for RTW) following MTBI[67]. In contrast, variables such as sex and age have not contributed to the prediction of RTW in most prior studies and were omitted from our model[23,51]. We included perceived injustice (IEQ) as a novel predictor of RTW because of its association with poor outcomes and disability in other medical populations[27,30,35] and because it may have an especially potent role in work-related injuries. Injury setting (i.e., workplace vs. non-workplace) was not included in the model because it was not related to RTW outcome in preliminary analyses (see Results). However, we were interested in potential interactions between injury setting and our other variables of interest, with the rationale that some variables (e.g., traumatic stress, perceived injustice) may be more salient and impactful for those injured at work. When introducing this interaction term, we also included the number of weeks between injury and the initial study assessment as a predictor in our analyses to control for the possible bias introduced because the workplace and non-workplace groups were not well-matched on the timing of initial study assessment. Predictors were entered into the logistic regression model in two steps. First, all candidate predictors were entered simultaneously (BC-PSI, PCL, IEQ, MSVT failure, co-occurring orthopedic injury, and self-rated prognosis). Second, the number of weeks between injury and the initial assessment and a single interaction term were added. The second step was repeated for each interaction term (workplace vs. non-workplace group x a predictor entered in the first step), one at time.

## Results

Demographics and injury characteristics are presented in Table 1. Workplace injuries comprised 45.1% of the final sample (n = 46). Participants with a workplace MTBI were somewhat younger than those who sustained a non-workplace MTBI, but this difference was not significant [t(98) = 1.71, p = .09]. The workplace MTBI group had a greater proportion of men [*Χ*^2^(1) = 7.38, p = .007] and they were less likely to have been educated beyond high school [56.5% vs. 80.4%, *Χ*^2^(1) = 6.78, p = .009]. Occupation differed between workplace and non-workplace MTBI [*Χ*^2^(6) = 19.90 p = .003], as did mechanism of injury [*Χ*^2^(5) = 32.71, p<.001]. Table 1 provides a breakdown of occupational categories and mechanisms of injury by group. There were no differences in rates of loss of consciousness, amnesia, and confusion/disorientation between workplace and non-workplace MTBI (*p*s>.05). The two groups also had a similar rate of a co-occurring orthopedic injury [*Χ*^2^(1) = 1.95, p = .16] and pre-injury mental health treatment [*Χ*^2^(1) = 1.52, p = .22].

**Table 1:** Demographic, injury, and initial assessment characteristics.

	Full Sample (n = 102)	Work Injury (n = 46)	Non-work Injury (n = 56)
**Demographics**			
Age, M (SD)	41.2 (11.7)	39.0 (11.8)	43.0 (11.4)
Sex, n (% female)	55 (53.9%)	18 (39.1%)	37 (66.1%)
Education level, n (%)			
Did not complete high school	8 (7.9%)	4 (8.7%)	4 (7.1%)
High School	23 (22.6%)	16 (34.8%)	7 (12.5%)
Some College	17 (16.7%)	8 (17.4%)	9 (16.1%)
Diploma	17 (16.7%)	8 (17.4%)	9 (16.1%)
Bachelor’s Degree	27 (26.5%)	8 (17.4%)	19 (33.9%)
Graduate Degree	10 (9.8%)	2 (4.3%)	8 (14.3%)
Occupation			
Manual Labor	20 (19.6%)	13 (28.3%)	7 (12.5%)
Skilled craft or trade	13 (12.7%)	9 (19.6%)	4 (7.1%)
Transport	4 (3.9%)	1 (2.2%)	3 (5.4%)
Sales and service	14 (13.7%)	7 (15.2%)	7 (12.5%)
Clerical	6 (5.9%)	0 (0%)	6 (10.7%)
Management or professional	23 (22.5%)	4 (8.7%)	19 (33.9%)
Other	22 (21.6%)	12 (26.1%)	10 (17.9%)
Pre-injury Mental Health Treatment	53 (52.0%)	27 (58.6%)	26 (46.4%)
**Injury Characteristics**			
Mechanism of Injury, n (%)			
Struck by object	30 (29.4%)	20 (43.5%)	10 (17.9%)
Motor vehicle crash	29 (28.4%)	3 (6.5%)	26 (46.4%)
Fall	28 (27.5%)	14 (30.4%)	14 (25.0%)
Sport	5 (4.9%)	0 (0%)	5 (8.9%)
Assault	5 (4.9%)	5 (10.9%)	0 (0%)
Other	5 (4.9%)	4 (8.7%)	1 (1.8%)
Loss of Consciousness, n (%)			
Witnessed LOC	18 (17.7%)	5 (10.9%)	13 (23.2%)
Unwitnessed LOC	23 (22.5%)	11 (23.9%)	12 (21.4%)
Unknown	12 (11.8%)	5 (10.9%)	7 (12.5%)
Denied	49 (48.0%)	25 (54.3%)	24 (42.9%)
Post-traumatic amnesia, n (%)	67 (65.7%)	33 (71.7%)	34 (60.7%)
Acute confusion/disorientation, n (%)	90 (88.2%)	42 (91.3%)	48 (85.7%)
Co-occurring orthopedic injury, n (%)	63 (61.8%)	21 (45.7%)	38 (67.9%)
**Initial Assessment Characteristics**			
Weeks to initial assessment, M (SD)	12.05 (6.3)	10.1 (5.5)	13.7 (6.4)
Primary care visits since injury, M (SD)	6.4 (4.4)	5.9 (3.9)	6.8 (4.7)
Self-Reported Treatment Utilization Since Injury, n (%)			
Emergency Department	78 (76.4%)	32 (68.6%)	46 (82.1%)
Specialist (e.g., neurologist)	39 (38.2%)	24 (52.2%)	15 (26.8%)
Physiotherapist	47 (46.1%)	19 (41.3%)	28 (50.0%)
Occupational Therapist	12 (11.8%)	4 (8.7%)	8 (14.3%)
Psychological therapy/counselling	10 (9.8%)	2 (4.3%)	8 (14.3%)
Massage Therapy	31 (30.4%)	7 (15.2%)	24 (52.2%)
Chiropractic treatments	12 (11.8%)	3 (13.0%)	9 (16.1%)
Acupuncture treatment	10 (9.8%)	3 (6.5%)	7 (12.5%)
Compensation status at initial assessment, n (%)			
No compensation	13 (12.7%)	1 (2.2%)	12 (21.4%)
Seeking/receiving administrative benefits	71 (69.6%)	45 (97.8%)	26 (46.4%)
Litigating	18 (17.6%)	0 (0%)	18 (32.1%)
Return to work status, n (%)			
Full return to work	11 (10.8%)	1 (2.2%)	10 (17.9%)
Partial return to work	16 (15.7%)	4 (8.7%)	12 (21.4%)
On leave	73 (71.6%)	40 (87.0%)	33 (58.9%)
Other	2 (2.0%)	1 (2.2%)	1 (1.8%)
Perceived Injustice; IEQ Total, M (SD)	20.8 (10.9)	22.7 (10.5)	19.2 (11.3)
Traumatic Stress; PCL-5, M (SD)	29.2 (17.0)	32.7 (17.9)	26.2 (15.9)
Brief Pain Questionnaire (BPQ), M (SD)	4.9 (3.3)	5.6 (3.5)	4.3 (3.0)
Post-Concussion Symptoms; BC-PSI, M (SD)	25.4 (12.8)	27.0 (13.4)	24.1 (12.2)
Performance Validity; MSVT Failures, n (%)	23 (22.5%)	9 (19.6%)	14 (25.0%)
Self-Prognosis Rating, n (%) (Note: n = 3 missing)			
Get better soon	14 (14.3%)	7 (16.3%)	7 (12.7%)
Get better slowly	60 (61.2%)	29 (66.4%)	31 (56.4%)
I don’t know	24 (24.5%)	7 (16.3%)	17 (30.9%)

The information in this table was collected between 2 and 26 weeks following injury (M = 12.06, SD = 6.3). Abbreviations: BC-PSI = British Columbia Postconcussion Symptom Inventory; IEQ = Injustice Experience Questionnaire; FABQ = Fear Avoidance Beliefs Questionnaire; M = mean; PCL-5 = PTSD Checklist for DSM-5; SD = standard deviation.

The workplace MTBI group had a shorter duration between their injury and the initial study assessment compared to the non-workplace MTBI group [t(99) = 2.92, p = .004, Cohen’s d = -0.60]. That is, they present for outpatient speciality MTBI care sooner. Time since injury was therefore used as a covariate in comparisons between groups on non-categorical variables (i.e., ANCOVA). Participants had seen their primary care physician an average of 6.4 times (SD = 4.4, Md = 5, IQR = 5–8) before the initial study assessment; there was no significant difference in the number of post-MTBI primary care visits between the workplace (M = 5.9, Md = 5) and non-workplace (M = 6.8, Md = 6) groups (Mann-Whitney U = 1120.0, p = .19). Additional self-reported healthcare utilization between the injury and initial study assessment can be seen in Table 1.

As expected, virtually all participants with workplace MTBI were off work and receiving administrative (wage loss) benefits at the time of the initial assessment. That is what would have prompted their referral for specialized MTBI treatment. Participants with non-workplace MTBI were more likely to have already returned to work in some capacity [*Χ*^2^(3) = 11.16, p = .011] by the initial assessment and were more likely to be seeking compensation through different means [*Χ*^2^(2) = 31.72, p<.001], such as personal injury litigation (see Table 1). In the initial assessment, the workplace MTBI group had more symptoms of traumatic stress [PCL-5, F(1, 98) = 4.04, p = .047, adjusted d = 0.42] when covarying for weeks since injury. There were no differences between workplace and non-workplace MTBI groups on perceived injustice [IEQ, F(1, 98) = 2.89, p = .09], overall post-concussion symptom severity (BC-PSI total score, F(1, 98) = 1.39, p = .24), or pain [BPQ, F(1, 97) = 2.75, p = .10] when covarying for weeks since injury. There were no group differences on MSVT failure rate [*Χ*^2^(1) = 0.43, p = .51] or recovery expectations [Self Prognosis; *Χ*^2^(2) = 2.81, p = .25].

Of the 102 participants who completed the initial assessment at clinic intake, 15 (14.7%) were lost to follow-up. Participants who were lost to follow-up did not differ from those who were retained with respect to age, sex, education (i.e., secondary vs. not), or race/ethnicity (Caucasian vs. non-Caucasian; *p*s>.05). A similar proportion of patients were lost to follow-up in the workplace and non-workplace MTBI groups [*Χ*^2^(1) = 0.48, p = .49]. The workplace and non-workplace MTBI groups did not differ on time between the injury and follow-up assessment [t(84) = 0.43, p = .67]. Similar to the initial assessment, the workplace and non-workplace MTBI groups differed in compensation status at follow-up [*Χ*^2^(3) = 25.71, p<.001].

Access to treatment is inherently different for patients inside vs. outside of the Worker’s Compensation system. Participants who sustained a non-workplace MTBI typically received only a group education session (100%), with a minority (<10%) receiving additional services in that clinic (e.g., physiatry consultation). Many of these participants reported accessing additional services in the community. In contrast, participants who sustained a workplace MTBI commonly received a brief neuropsychological assessment (100%), medical examination (85.7%), multi-day interdisciplinary assessment (80.9%), multi-week rehabilitation program (68.6%), gradual RTW transition support (57.1%), and a job site visit (40.0%).

Outcomes obtained from the follow-up telephone assessment are shown in Table 2. At follow-up, there were no differences in RTW status between the workplace and non-workplace MTBI groups [*Χ*^2^(2) = 0.16, p = .92; Cramer’s V = 0.04]. Collapsing those with full RTW and partial RTW into a single “any RTW” group did not alter the results. At follow-up, there were no group differences in post-concussion symptoms (BC-PSI, t(84) = -0.10, p = .92, d = 0.07), functional disability (WHODAS, t(85) = 0.55, p = .59, d = -0.12), or pain (BPQ, t(85) = -0.28, p = .78, d = 0.08) between groups. Similar proportions of both groups were operationalized as having mild ICD-10 postconcussional syndrome [*Χ*^2^(1) = 0.23, p = .63; V = 0.05] and moderate ICD-10 postconcussional syndrome [*Χ*^2^(1) = 0.35, p = .56; V = 0.06]. Using a more conservative definition of complete RTW (i.e., full RTW and LEAPS score 0–5[57]) did not reveal group differences. A similar proportion of participants in the workplace and non-workplace MTBI groups fully returned to work with normal productivity [*Χ*^2^(1) = 0.02, p = .89].

**Table 2:** Follow-up characteristics.

	Follow-up Sample (n = 87)	Work Injury (n = 38)	Non-work Injury (n = 49)
**Follow-up Characteristics**			
Lost to follow-up, n (%)	15 (14.7%)	8 (17.4%)	7 (12.5%)
Weeks from Injury to Follow-Up, M (SD)	31.6 (6.1)	31.2 (6.2)	31.8 (6.0)
Compensation status at follow-up, n (%)			
No compensation	40 (46.0%)	24 (63.2%)	16 (32.7%)
Seeking/receiving administrative benefits	23 (26.4%)	14 (34.8%)	9 (18.4%)
Litigating	33 (26.4%)	0 (0%)	23 (46.9%)
Claim Settled	1 (1.1%)	0 (0%)	1 (2.0%)
*Primary Outcome*			
Return to work status, n (%)			
Full return to work	37 (42.5%)	17 (44.7%)	20 (40.8%)
Partial return to work	16 (18.4%)	7 (18.4%)	9 (18.4%)
No return to work	34 (39.1%)	14 (36.8%)	20 (40.8%)
*Secondary Outcomes*			
Post-Concussion Symptoms; BC-PSI, M (SD)	20.1 (13.2)	20.2 (13.0)	19.9 (13.5)
Brief Pain Questionnaire (BPQ), M (SD)	4.5 (3.7)	4.7 (3.4)	4.4 (4.0)
ICD-10 Postconcussional Syndrome, n (%)			
Based on Mild+ Symptoms	73 (83.7%)	31 (81.6%)	41 (85.4%)
Based on Moderate+ Symptoms	55 (64.9%)	23 (60.5%)	32 (66.7%)
WHODAS 2.0 Total Score, M (SD)	25.8 (9.6)	25.2 (8.7)	26.3 (10.3)
Return to work status (alternate definition)			
Full RTW with LEAPS = 0–5	20 (23.0%)	9 (23.7%)	11 (22.4%)
No RTW/Partial RTW/Full RTW with LEAPS≥6	67 (77.0%)	29 (76.3%)	38 (77.6%)

Abbreviations: BC-PSI = British Columbia Postconcussion Symptom Inventory; ICD-10 = International Statistical Classification of Diseases and Related Health Problems, 10^th^ Revision; M = mean; RTW = Return to work; SD = standard deviation; WHODAS = World Health Organization Disability Assessment Schedule, 2.0

As shown in Table 3, greater perceived injustice, greater post-concussion symptoms, and answering “don’t know” when symptoms would resolve (compared to “get better soon”) were significantly related to delayed RTW in unadjusted (single predictor) regression models. The logistic regression with all predictor variables (step 1) was well calibrated [Hosmer and Lemeshow *Χ*^2^(7) = 5.58, p = .59] and statistically significant [*Χ*^2^(7) = 22.41, p = .002]. Prediction accuracy was fair (Nagelkerke R^2^ = .31; area under the receiver operating curve = .78, p<.001). In this model, post-concussion symptoms and traumatic stress symptoms were statistically significant, such that greater post-concussion symptoms and less traumatic symptoms were associated with not working at follow-up. These results did not change in Step 2, when time between injury and initial assessment were entered into the model. Further, none of the ‘injury setting-by-predictor’ interaction terms were statistically significant, indicating that none of the candidate risk factors were differentially related to RTW outcome in the workplace vs. non-workplace groups.

**Table 3:** Logistic regression results of return to work (RTW; any work vs. no work) status at follow-up.

	Odds ratio (95% confidence interval)		
Variable	Unadjusted	Step 1: Adjusted	Step 2: Weeks_injury-initial assessment_ and Interaction (Adjusted)
Weeks between injury and initial assessment	1.10 (1.01–1.19*)	-	-
Traumatic Stress (PCL-5)	1.01 (0.98–1.04)	93 (0.87–0.980.)*	0.92 (0.86–0.98)*
Interaction_Workplace_	-	-	1.00 (0.97–1.03)
Post-Concussion Symptoms (BC-PSI)	1.06 (1.02–1.10)*	1.10 (1.03–1.17)*	1.11 (1.03–1.19)*
Interaction_Workplace_	-	-	1.01 (0.97–1.05)
Perceived Injustice (IEQ)	1.05 (1.01–1.10)*	1.07 (1.00–1.16)	1.08 (1.00–1.17)
Interaction_Workplace_	-	-	1.00 (0.95–1.04)
Self-Rated Prognosis			
Better soon vs. Better slowly	0.97 (0.54–1.74)	0.74 (0.34–1.60)	0.63 (0.22–1.85)
Interaction_Workplace_	-	-	1.21 (0.31–4.68)
Better soon vs. Don’t know	2.19 (1.04–4.64)*	1.38 (0.58–3.29)	1.05 (0.34–3.23)
Interaction_Workplace_	-	-	1.32 (0.20–8.88)
Co-occurring orthopedic injury	0.59 (0.23–1.47)	0.87 (0.27–2.79)	0.82 (0.22–3.02)
Interaction_Workplace_	-	-	1.26 (0.56–2.86)
MSVT Failure	2.34 (0.85–6.45)	1.74 (0.41–7.29)	1.00 (0.18–5.74)
Interaction_Workplace_	-	-	2.93 (0.28–31.06)

* indicates the Odds Ration (OR) was statistically significant at p = .05.

Abbreviations: BC-PSI = British Columbia Postconcussion Symptom Inventory; IEQ = Injustice Experience Questionnaire; MSVT = Medical Symptom Validity Test; PCL-5 = PTSD Checklist for DSM-5.

## Discussion

There are important differences between the pre-injury characteristics, mechanisms of injury, health care services, and recovery trajectories between people who sustain an MTBI in sport, military, and civilian trauma settings[68,69]. Prior studies examining traumatic injuries of all kinds have reported that people who are injured in workplace accidents are at elevated risk for poor outcomes[7,70]. The primary aim of the present study was to compare patients with workplace vs. non-workplace MTBI. We studied patients who were referred to specialty care because of atypical recovery following MTBI. Almost all participants met ICD-10 symptom criteria for postconcussional syndrome during the study period, and their symptom reporting was likely influenced by factors separate from neurotrauma (such as traumatic stress, persistent bodily pain, and exaggeration). When interpreting the results of this study, it is important to appreciate that the sample was specialty clinic-referred, and generally slow-to-recover, highly symptomatic, with disproportionately high rates of risk factors for poor outcome. We hypothesized that patients injured at work would be a distinct subgroup, with more adverse risk profiles and lower rates of RTW compared to patients who were injured in other settings. We expected that our findings would support a stratification that may help explain the heterogeneity of MTBI outcomes. Overall, our study did not demonstrate important differences between workplace and non-workplace related MTBIs in these specialty clinic samples.

We found similarities and differences between patients with persistent symptoms following workplace vs. non-workplace MTBI. The two groups similarly accessed primary care during the acute post-injury period, but patients with workplace MTBI presented for outpatient speciality care a few weeks faster, on average. This difference is not surprising because those with a Worker’s Compensation claim are assigned a case manager whose role it is monitor the worker’s recovery and to initiate referrals for treatment. For people who sustain an MTBI in other settings, navigating health care services are generally left to the patient themselves and/or their primary care provider. Consistent with the broader literature on musculoskeletal injuries[21–24], patients with workplace MTBI were more likely to be male blue-collar workers without a Bachelor’s or graduate degree. Falls were a common mechanism of injury in both groups, but patients with workplace injuries most frequently sustained their MTBI by being struck with an object, whereas motor vehicle accidents were the most frequent cause of non-workplace MTBI.

At clinic intake (2–3 months post injury), there were no significant group differences in post-concussion symptoms, perceived injustice, or expectations for recovery. The only difference was that workplace MTBI was associated with greater PTSD symptoms. This could, in part, be related to the differences in mechanisms of injuries (e.g., more assaults, fewer sport injuries). Despite differences in compensation access (patients with Worker’s Compensation Claims receive administrative benefits but cannot sue whereas patients with non-workplace injuries may not have access to administrative benefits but often can seek compensation through personal injury litigation), the rate of suspected exaggeration on neuropsychological testing (performance validity test failure) was similar and fairly high in both groups (20–25%).

The workplace and non-workplace MTBI groups were indistinguishable at follow-up, 6–7 months post injury. A very similar number had returned to work in a partial (18–19%) or full (37–41%) capacity. They also reported a similar burden of residual post-concussion symptoms (on the BC-PSI), functional disability (on the WHODAS), and pain (on the BPQ). An absence of differences in post-acute outcome is in keeping with the finding that workplace MTBI was associated with both favorable (male sex) and unfavorable (low education and job decision-making latitude) prognostic factors, and similar psychosocial profiles to participants with non-workplace MTBI at clinic intake, with the exception of more PTSD symptoms. However, the absence of differences in post-acute outcome might be surprising when considering that patients with workplace MTBI received more comprehensive and coordinated rehabilitation services. It is difficult to draw conclusions about why these two workplace and non-workplace MTBI groups ended up looking similar at follow-up. It may be that the workplace MTBI group was at greater risk of poor outcome due to unmeasured variables, and the timely and extensive rehabilitation they received offset that increased risk. It is also possible that both groups were on a similarly poor recovery trajectory, and the health services they received did not substantially influence their trajectories. We entertained that the workplace and non-workplace MTBI groups may have achieved similar outcomes via different pathways, and so also sought to examine whether certain risk factors for delayed RTW would be stronger in the workplace MTBI group. In single predictor models, perceived injustice, post-concussion symptoms, and having uncertain (vs. optimistic) recovery expectations at the time of clinic intake were associated with lower likelihood of returning to work 4–5 months later (i.e., by 6–7 months following injury). After adjusting for all variables in the model, only greater post-concussion symptoms were associated with a lower likelihood of RTW at follow-up. This is consistent with previous studies[23,51] demonstrating that symptom burden is predictive of a protracted recovery. Additionally, the multivariate model showed an association between *lower* traumatic stress symptoms and worse outcome. We suspect that this finding is an artefact of statistical suppression. Traumatic stress was not associated with RTW in unadjusted (single predictor) modeling but was highly correlated with post-concussion symptoms (r = .60). The collinearity of these measures likely enhanced the relationship between post-concussion symptoms and the outcomes (i.e., OR 1.06 to 1.10) and suppressed the relationship between traumatic stress and the outcome (i.e., 1.01 to 0.93). Contrary to our hypotheses that certain risk factors would be differentially associated with the workplace MTBI group, there were no statistically significant interactions effects.

This study has several noteworthy limitations. First, the workplace and non-workplace MTBI groups were not matched on the timing of their first assessment. This was an observational study and our two groups had inherently different access to healthcare. We employed a covariate adjustment for time since injury wherever possible. Second, we had very limited information on the screened participants who did not enroll in this study, precluding the analyses necessary to understand the presence and impact of selection bias. Third, as with all longitudinal studies, there was attrition. The rates of attrition were similar in both MTBI groups, and those who were lost to follow-up had similar demographics compared to those who were retrained. It seems unlikely that attrition biased the group comparisons of primary interest, workplace vs. non-workplace. Fourth, injury characteristics were assessed via self-report during a structured interview. Medical records were not available to review. Although self-reported data has limitations[71,72], the primary hypotheses regarding differences between the workplace and non-workplace MTBI groups should not be affected by these limitations. Fifth, the workplace and non-workplace MTBI groups differed on their treatment utilization following clinic intake. This imbalance is inherent to the health care system. Sixth, it is possible that we did not find group differences in outcomes because of sample size and statistical power limitations. However, the small magnitudes of the observed effect sizes suggest that large differences between work and non-work MTBIs are unlikely. Lastly, the follow-up period was not long enough to observe the outcome of interest (RTW) in large proportion of the sample, which precluded more powerful (time-to-event) statistical modeling techniques.

In summary, despite systemic differences in compensation and health care access, the workplace and non-workplace MTBI groups were similar at clinic intake and indistinguishable at follow-up, 6–7 months following injury. The workplace MTBI group had different demographic profiles (i.e., more men, lower education levels, fewer managerial/professional jobs) and greater traumatic stress symptoms at the initial assessment, but these differences did not translate into measurable divergence on any outcome measure. There was no evidence for risk factors for delayed RTW being more or less relevant to patients with workplace MTBI vs non-workplace MTBI. At the follow-up assessment 6–7 months after MTBI, a minority of individuals had achieved their pre-injury work productivity levels, and the large majority continued to meet symptom criteria for at least mild ICD-10 postconcussional syndrome. It is important to interpret these findings in the context of our study design. Participants in both groups were clinic-referred, slow-to-recover, highly symptomatic, and had disproportionately high rates of risk factors for poor outcome. They were not representative of all people who sustain an MTBI. However, the present findings may generalize to other outpatient clinic settings where patients with MTBI are commonly seen. This study highlights the need for intervention research to improve functional outcomes in patients with MTBI who present for outpatient care. An improved characterization of risk factors could inform a personalized medicine approach, whereby injured workers are matched to therapies designed to target the source of their heightened risk.

## Supporting information

S1 Dataset Excel spreadsheet containing the study dataset. (XLSX)
